# A rare cause of gastro-intestinal hemorrhage in a patient with a Roux-en-Y gastric bypass

**DOI:** 10.1093/gastro/gou056

**Published:** 2014-08-23

**Authors:** Richard H. Cartabuke, Paresh P. Mehta, Kevin El-Hayek, J. Michael Henderson, Carol A. Burke

**Affiliations:** ^1^Department of Internal Medicine, Cleveland Clinic, Cleveland, Ohio, USA,; ^2^Department of Gastroenterology and Hepatology, Cleveland Clinic, Cleveland, Ohio, USA and; ^3^Department of General Surgery, Cleveland Clinic, Cleveland, Ohio, USA

**Keywords:** gastro-intestinal bleeding, superior mesenteric venous thrombosis, Roux-en-Y gastric bypass

## Abstract

This case illustrates a rare cause of gastro-intestinal bleeding following bariatric surgery. Though it is essential to rule out common causes of variceal formation accompanied by intermittent, profuse bleeding, there should be a high degree of suspicion of this rare etiology in patients who have previously undergone alteration of their anatomy, especially Roux-en-Y gastric bypass (RYGB). The case emphasizes the need for a multidisciplinary medical-surgical team in evaluating and treating patients who present with complex intra-abdominal pathology.

## INTRODUCTION

Obscure gastro-intestinal bleeding in patients with altered gastro-intestinal anatomy poses both a diagnostic and therapeutic challenge. As bariatric surgery becomes increasingly commonplace, complications will be observed more frequently. Herein, we report an unusual cause of recurrent, small intestinal variceal hemorrhage due to complications from a Roux en Y gastric bypass.

## CASE PRESENTATION

A 53-year-old year old woman was admitted to our hospital for overt gastro-intestinal hemorrhage with associated hemodynamic instability and after multiple blood transfusions. Her previous surgical history included a laparoscopic antecolic-antegastric Roux-en-Y gastric bypass (RYGB; 150 cm Roux limb) for morbid obesity in 2010. The patient had lost 72% of her excess body weight, reflecting an absolute reduction of 105 pounds. 3 years after her RYGB the patient underwent laparoscopic reduction of an internal hernia which occurred secondary to a mesenteric defect. Prior to transfer to our hospital, she had three episodes of melena and hematochezia over a period of three months, no abnormalities being detected during two technetium-99 red blood cell scans, three colonoscopies, two esophago-gastroduodenoscopies (EGD), and a mesenteric angiogram. She reported no use of non-steroidal anti-inflammatory drugs (NSAID) or oral anticoagulants prior to her initial presentation.

**Figure 1:**
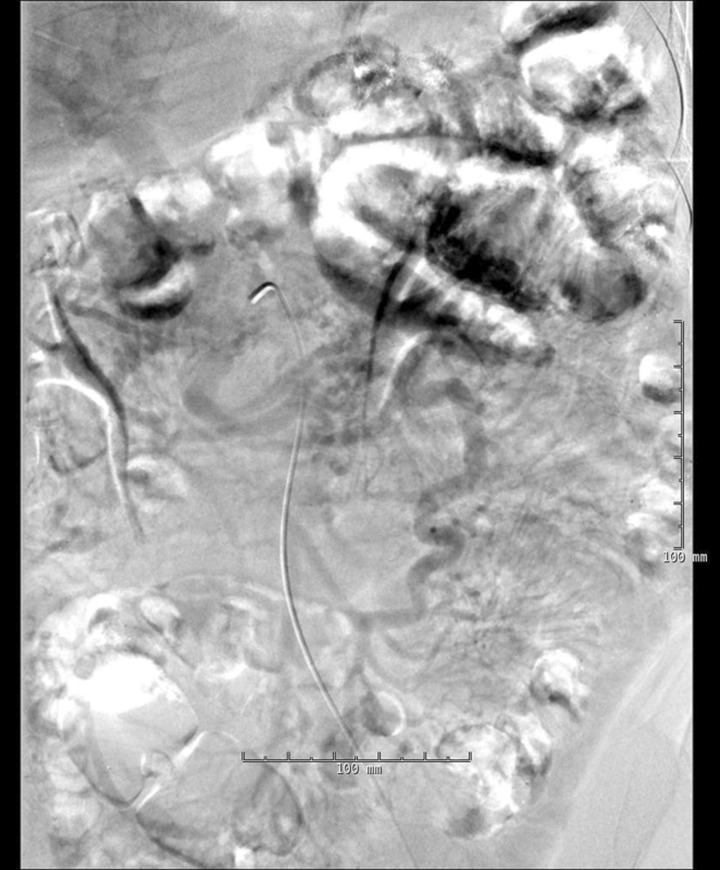
Computed tomography angiography of the superior mesenteric artery was filmed into the portal venous phase. There was filling of the ileocolic vein, as well as of the jejunal branches. At the confluence of the jejunal branches with the ileocolic vein, there was an abrupt occlusion at the ligament of Treitz, suggestive of the operative finding of volvulus. The normal expected proximal most portion of the superior mesenteric vein, along with its drainage into the splenic vein, is absent.

**Figure 2:**
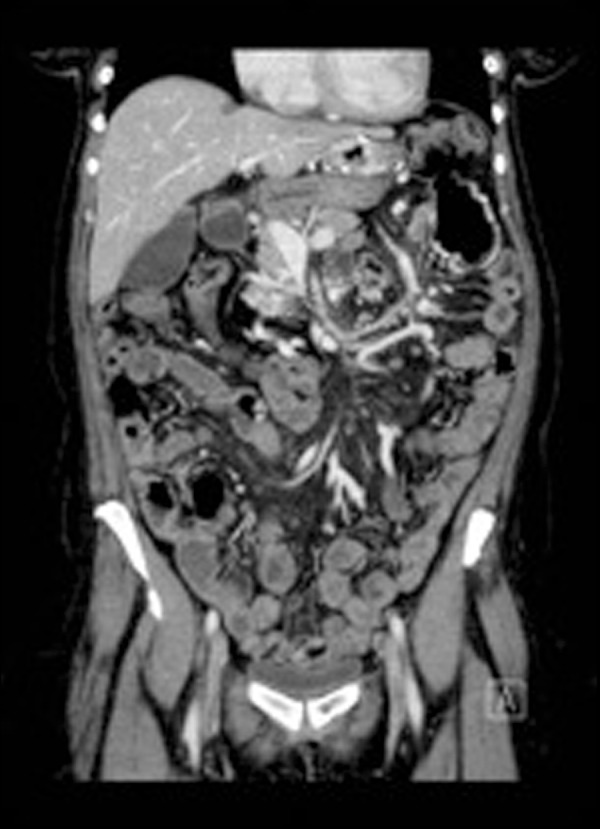
Computed tomography of the abdomen and pelvis: the superior mesenteric vein (SMV) is obliterated, with no visible normal SMV present. Instead, the veins of the small intestine and colon drain into collateral pathways, indicating chronic venous occlusion.

**Figure 3:**
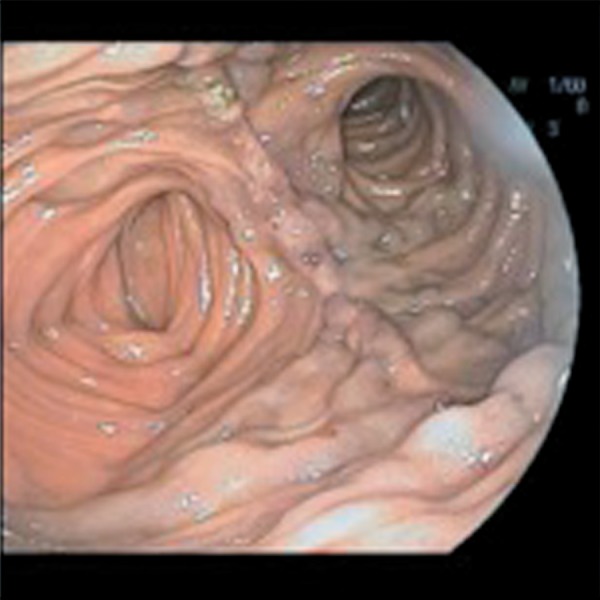
Large, non-bleeding varices at the jejunojejunal anastomosis.

Upon admission, the patient's blood pressure was 162/69, pulse 79 beats per minute, respiration rate 20 breaths per minute, and temperature of 36.8°C. Her abdomen was soft, non-distended and non-tender, with well-healed abdominal scars. Her hemoglobin was 7.8 g/dL with an otherwise normal complete blood count and comprehensive metabolic profile. An EGD revealed a normal esophagus, gastric pouch with intact staple line, and jejunum as far as could be examined using an upper endoscope. Colonoscopy of the cecum revealed a medium-sized, non-bleeding vein in the transverse colon. Video capsule endoscopy of the small bowel was normal. Both angiogram and computed tomography of the abdomen revealed an abrupt cut-off of the superior mesenteric vein (SMV), with the development of venous collaterals draining the small intestine and colon (Figures 1 and 2). Results of a liver vascular ultrasound were unremarkable. Antegrade balloon enteroscopy demonstrated large, non-bleeding varices at the jejunojejunal (JJ) anastomosis (Figure 3). There were also signs of varices at the gastrojejunal (GJ) anastomosis that had not initially been observed during the above-mentioned endoscopy. The diagnosis was superior mesenteric venous thrombosis with secondary Roux limb varices at the gastrojejunal and jejunojejunal anastomoses.

Prior to surgery, consideration was given to the placement of a mesenteric-systemic shunt but this was rejected, since the venous hypertension was postulated to be the result of mechanical obstruction that required correction. The patient underwent exploratory laparotomy for definitive management. The intra-operative findings included varices affecting the entire Roux limb as well as the gastrojejunostomy and jejunojejunostomy. Additional findings included a 180° volvulus of the small bowel at the root of the mesentery, which was probably the cause of the SMV. The bowel was untwisted to relieve the volvulus. The Roux limb was resected from the level of the jejunojejunostomy to the gastrojejunostomy. The patient's anatomy was then restored with a gastrogastrostomy and jejunojejunostomy. Her post-operative course was uneventful and the patient was discharged home seven days after surgery. After three months, the patient showed no further signs of bleeding.

## DISCUSSION

Bariatric surgery is increasing steadily all over the world, with women representing the majority of patients (70%). Laparoscopy has become the technique of choice for RYGB. One of the main disadvantages of laparoscopic—compared with open gastric—bypass is the increased incidence of internal hernia, due to the difficulty of properly identifying and closing mesenteric defects. This is also complicated by the specific operative approach, as well as individual surgeons' preference to close all defects to prevent post-operative internal herniation [7]; such defects include jejunojejunostomy, mesenteric defects, transverse mesocolon defects, and Petersen’s defect (between the Roux limb and the transverse mesocolon). The antecolic procedure removes defects of the mesocolon, considered the most common site of internal herniation following the retrocolic approach. However, the antecolic approach usually creates another defect termed ‘pseudo-Petersen’s defect'. If this defect is quite large, there is a potential increase in the risk of internal hernia, especially when the mesentery is divided to release tension from the gastrojejunal anastomosis. It has been postulated that the weight loss seen in these very obese patients—typically occurring some months after bariatric surgery—causes rapid reduction of the intraperitoneal fat which, in turn, leads to a subsequent enlargement of the surgically created mesenteric defect and a consecutive loosening of the mesenteric sutures [1].

A known *sequelea* of internal hernia after laparoscopic RYGB is small bowel obstruction, with a reported incidence of 0.9–5.0% [7]. The clinical presentation of an internal hernia ranges from minor abdominal pain requiring elective repair, to profound peritonitis—due to massive segments of ischemic/necrotic bowel—requiring resection. A thorough search of the literature reveals considerable information on obstructive and ischemic pathology—particularly as it relates to acute mesenteric venous thrombosis—but none covering the presentation of gastro-intestinal bleeding [2–5].

Management in this case was focused on mitigation of the high-pressure variceal system created by the chronic venous obstruction, probably resulting from abutment of the displaced small bowel loops around the superior mesenteric vessels. Vascular intervention was considered in the form of a variceal–systemic shunt, but this was not felt to be a long-term solution option. Data are scarce and limited to case reports; however, endovascular recanalization and portal reconstruction, as well as mesentero-systemic shunting, have been employed [6]. The Roux limb was resected *en bloc* and a gastrogastrostomy and jejunojejunostomy was created, thus effectively reversing the patient's gastric bypass anatomy in an attempt to decrease her outflow high-pressure gradient from the Roux limb to both the gastrojejunostomy and jejunojejunostomy. The patient had experienced no further episodes of bleeding 10 months after the initial procedure.

**Conflict of interest:** none declared.
